# Antimicrobial activity of PVP coated silver nanoparticles synthesized by *Lysinibacillus varians*

**DOI:** 10.1007/s13205-016-0514-7

**Published:** 2016-09-07

**Authors:** Divya Bhatia, Ashwani Mittal, Deepak Kumar Malik

**Affiliations:** 1 University College, Kurukshetra University, Kurukshetra, India; 2 University Institute of Engineering and Technology, Kurukshetra University, Kurukshetra, India

**Keywords:** Silver nanoparticles, Biosynthesis, Characterization, Antimicrobial

## Abstract

Emergence of resistant microbes to conventional antibiotics and increased emphasis on health-care costs has raised the concern for the development of new effective antimicrobial reagents. Silver nanoparticles being an excellent broad-spectrum antibacterial agent could be considered as a suitable alternative for existing antibiotic. This study demonstrates the extra-cellular synthesis of stable silver nanoparticles using supernatant of *Lysinibacillus varians*. The synthesized silver nanoparticles were characterized by using UV–visible spectrum analysis, X-ray diffraction, Transmission electron microscopy (TEM) and FT-IR analysis. The synthesized silver nanoparticles showed a peak around 420 nm. TEM analysis revealed that the size of silver nanoparticles was in the range of 10–20 nm. Silver nanoparticles carry a charge of −39.86 mV, which confirmed the stability of silver nanoparticles. The biologically synthesized silver nanoparticles showed antimicrobial activity against Gram-positive, Gram-negative bacteria and fungi. Therefore, the current study reveals an efficient and eco-friendly synthesis of silver nanoparticles by *L. varians* with potent antimicrobial activity.

## Introduction

Nanotechnology is an interdisciplinary field which impacts significantly all aspects of human’s life (Mohanpuria et al. 2008; Liu et al. 2011). This field deals with the synthesis and applications of nanomaterials in various areas (Duran et al. 2005). Metal nanoparticles exhibit unique properties, which depend on their size, shape, composition, and dielectric surroundings (Hubenthal et al. 2005; Chen and Goodman 2004; Jain et al. 2006). Ag-NPs have numerous applications in the field of diagnostics, textile, catalysis, optics, photography, electronics and food industry (Rai et al. 2009). Silver nanoparticles have been used as antimicrobial agents in surgically implanted catheters, surgical bandages, eye treatment, dental hygiene, bone substitute biomaterials, disinfecting medical devices/home appliances and textile coatings (Bosetti et al. 2002; Cho et al. 2005; Gupta and Silver 1998; Jain and Pradeep 2005; Li et al. 2008). Microbial synthesis of metal nanoparticles can take place either intracellularly or extracellularly (Ahmad et al. 2007; Jain et al. 2011; Kalishwaralal et al. 2010; Pugazhenthiran et al. 2009; Saifuddin et al. 2009). Intracellular synthesis of nanoparticles requires additional steps to release the synthesized nanoparticles (Kalimuthu et al. 2008). At the same time extracellular biosynthesis is cheap and requires simpler downstream processing. This paper reports the extracellular biosynthesis of silver nanoparticles with their potent antimicrobial activity.

## Materials and methods

### Isolation and characterization of bacteria

All the chemicals used were of analytical grade purchased from Himedia Laboratories Pvt. Ltd., India. The isolation of bacteria for silver nanoparticle synthesis was carried out by serial dilution method from metal contaminated soil (Baker and Shreedharmurthy 2012). The serially diluted soil sample was spreaded over nutrient agar plates. The plates were incubated at 30 °C for 48 hours. The isolated colonies were further sub-cultured on nutrient agar supplemented with 1 mM silver nitrate (AgNO_3_) for screening of bacterial culture capable to grow in the presence of silver nitrate. The isolated bacterial culture capable to synthesize silver nanoparticles synthesis was characterized by 16S rRNA sequencing from the Institute of Microbial Technology, Chandigarh (India).

### Extracellular synthesis of silver nanoparticles

The synthesis of AgNPs was carried out by using extracellular method (Kalishwaralal et al. 2008). The isolated bacterial culture DNP 10 was inoculated in basal salt media (BSM) for 48 hours at 30 °C under shaking conditions of 220 rpm. The composition of BSM was (in g/L) (NH_4_)_2_SO_4_, 1.0; K_2_HPO_4_, 0.1; MgSO_4_, 0.2; FeSO_4_·7H_2_O, 0.001; NaCl, 1.0; Na_2_MoO_4_, 0.0033. After incubation, the broth culture was centrifuged at 8000 rpm for 20 minutes. The supernatant was used for the synthesis of AgNPs. Three Erlenmeyer flasks, first containing supernatant with 2 mM silver nitrate in ratio of 1:1 with 0.1 % polyvinyl pyrrolidone (PVP) as a stabilizing agent, second containing only supernatant and third containing only silver nitrate solution were incubated under bright conditions for 30 minutes. The appearance of brown colour was an indicator for the synthesis of AgNPs. The extracellular synthesis of AgNPs was monitored by observing change in the colour of the culture supernatant from transparent to brown.

### Characterization of silver nanoparticles

The silver nanoparticle synthesis was further confirmed by UV–Vis spectroscopy analysis in the range of 300–900 nm by using a UV-2550 PC UV–Vis spectrometer (Shimadzu, UV Pharma spec 2550 with a resolution of 0.72 nm). The deionized water was used as blank for all measurements. The Silver nanoparticles were separated by centrifugation at 10,000 rpm for 40 minutes at 4 °C. The pellet was washed five times with distilled water, air dried and used for further characterization. The purified silver nanoparticles were characterized by X-ray diffraction **(**XRD), for phase structure and exact material identification. The size and shape of silver nanoparticles was analyzed by TEM using Hitachi (H-7500) with an electron kinetic energy of 120 kV at CIL, Panjab University, Chandigarh. The size-distribution profile and zeta potential of silver nanoparticles was studied by using dynamic light scattering (DLS) measurements conducted with a Microtrac Nanotrac Wave Particle Size, Zeta Potential analyzer. The FTIR spectrum was studied by using FT-IR spectrophotometer (Horizon ABB) between the spectral range of 4000–400 cm^−1^ at a scan speed of 16 cm/s.

### Analysis of Antimicrobial activity of AgNPs

The antimicrobial activity of biologically synthesized silver nanoparticles was tested against ten different test pathogens (seven bacteria and three fungi). The test micro-organisms used were *Escherichia coli* (MTCC No 40)*, Bacillus subtilis* (MTCC No 441)*, Pseudomonas aeruginosa* (MTCC No 424)*, Pseudomonas fluorescens* (MTCC No 1748), *Staphylococcus aureus* (MTCC No 87), *Streptococcus mutans* (MTCC No 497), *Streptococcus pyogenes* (MTCC No 1924)*, Fusarium graminearum* (MTCC No 2089)*, Candida albicans* (MTCC No 3017) and *Candida glabrata* (MTCC No 3019). The cultures were procured from MTCC, Institute of Microbial Technology, Chandigarh. The antibacterial activity of synthesized AgNPs was checked by agar well diffusion assay with some modification (Perez et al. 1990). The nutrient agar plates were spreaded with 100 µL inoculum (1.5 × 10^8^ CFU/mL) of each selected pathogen (in triplicates). Three wells were made with sterile borer into inoculated agar plates. The biologically synthesized silver nanoparticles (70 µL) were poured into one well of inoculated plates. In other two wells, equal volume of silver nitrate and culture supernatant was poured. These two wells were considered as control. After incubation at 35 °C for 24 hours, zone of inhibition was measured and recorded as mean ± SD of the triplicate experiment.

### Minimum inhibitory concentration (MIC)

Minimum inhibitory concentration of silver nanoparticles against test pathogens (*E. coli*, *Bacillus subtilis*, *P. aeruginosa*, *S. aureus* and *C. albicans*) was determined by agar well diffusion assay method with some modifications (Perez et al. 1990). To examine the minimum inhibitory concentration of silver nanoparticles, different concentrations of silver nanoparticles (375, 187.5, 93.75, 46.85, and 23.45 μg) were prepared. Two fold serial dilutions of silver nanoparticles were prepared in deionized water. The nutrient agar plates were inoculated with 100 µL of standardized inoculum (1.5 × 10^8^ CFU/mL) of each selected pathogen (in triplicates) and spreaded with sterile swabs. The wells of 8 mm size were made with sterile borer into agar plates containing the inoculums. The lower portion of wells was sealed with a little molten agar medium. Next, the serially diluted silver nanoparticles (50 µL) were poured into different wells of inoculated plates. The plates were incubated at 30 °C for 24 hours. The lowest concentration of silver nanoparticles that inhibits the growth of test pathogens was considered as the minimum inhibitory concentration (MIC).

## Results

The soil sample from metal contaminated site was used for the isolation of bacteria capable to synthesize silver nanoparticles. After screening for their growth over nutrient agar plates containing silver nitrate (AgNO_3_), the bacterial isolate DNP10 was selected for further investigation. Antimicrobial property of silver nitrate is well known since ancient times. It is assumed that bacteria capable to grow in the presence of silver nitrate will have higher chances to produce silver nanoparticles by reducing silver nitrate. However, there is no direct correlation between the growth of isolated bacteria over silver nitrate containing nutrient agar plates and ability to synthesize the silver nanoparticles.

The 16S rDNA sequence data of the DNP10 was subjected to BLAST analysis. The result showed 100 % identity to reported 16S rDNA sequence of *Lysinibacillus varians*. The 16S rDNA sequence of the bacterial isolate DNP10 was submitted to NCBI under the accession number KX011876.

**Fig. 1:**
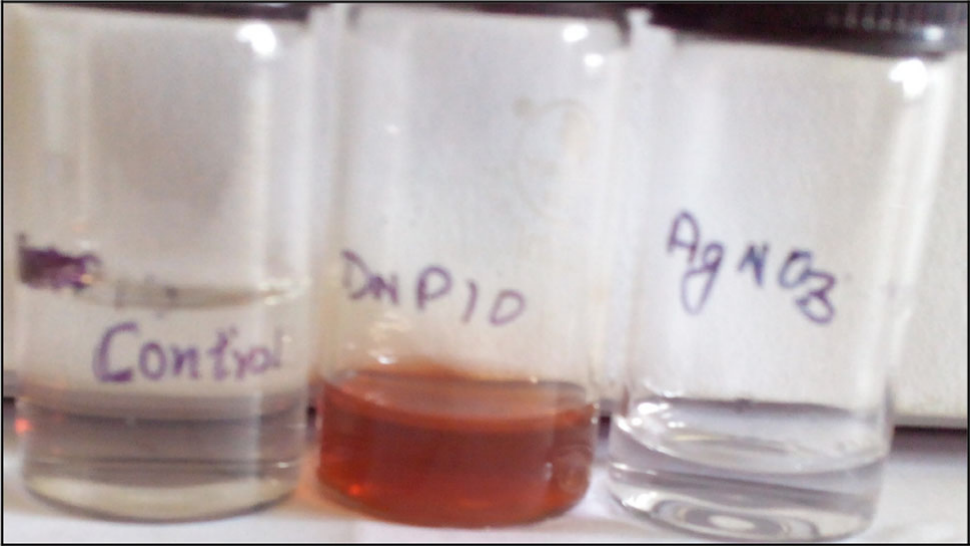
Extracellular synthesis of silver nanoparticles by bacterial isolate DNP 10

**Fig. 2:**
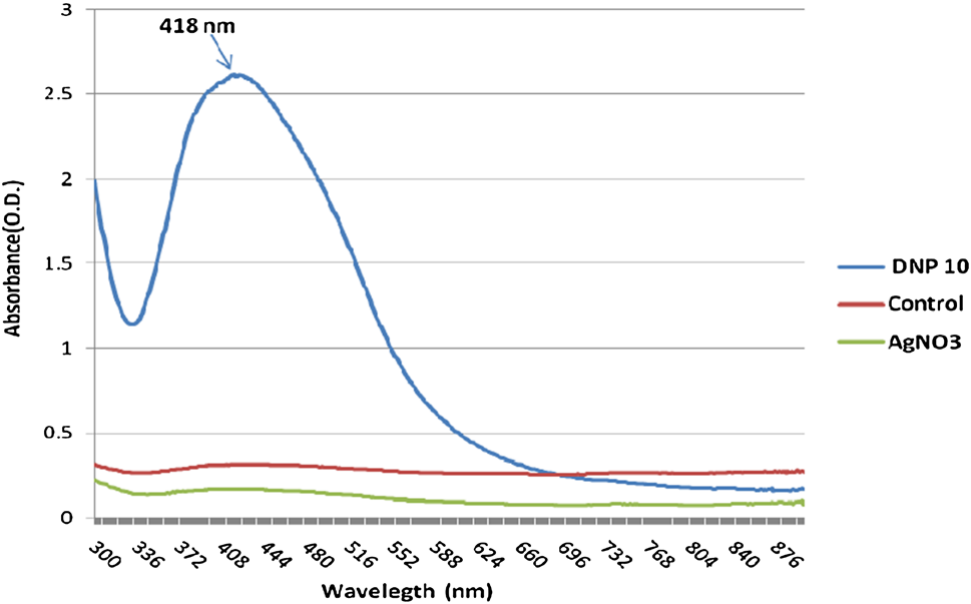
UV Visible Spectrum analysis of biologically synthesized AgNPs

The synthesis of silver nanoparticles was observed by the change in colour of culture supernatant in the presence of AgNO_3_. The flask containing DNP 10 cell free supernatant with silver nitrate (2 mM) and PVP showed colour change from transparent to light yellow within 2 minutes of incubation. The change in colour from light yellow to dark brown was observed after 20 minutes of incubation period as shown in Fig. 1. The two other flasks (control), one contained only supernatant and the other contained only silver nitrate showed no colour change. This suggests that the colour change observed in case of bacterial supernatant with silver nitrate was due to the formation of silver nanoparticles. The biologically synthesized silver nanoparticles were characterized by UV–Visible spectroscopy as shown in Fig. 2. A characteristic sharp peak around 418 nm wavelength was observed, which confirmed the presence of AgNPs. This is the characteristic wavelength for the synthesis of AgNPs.

**Fig. 3:**
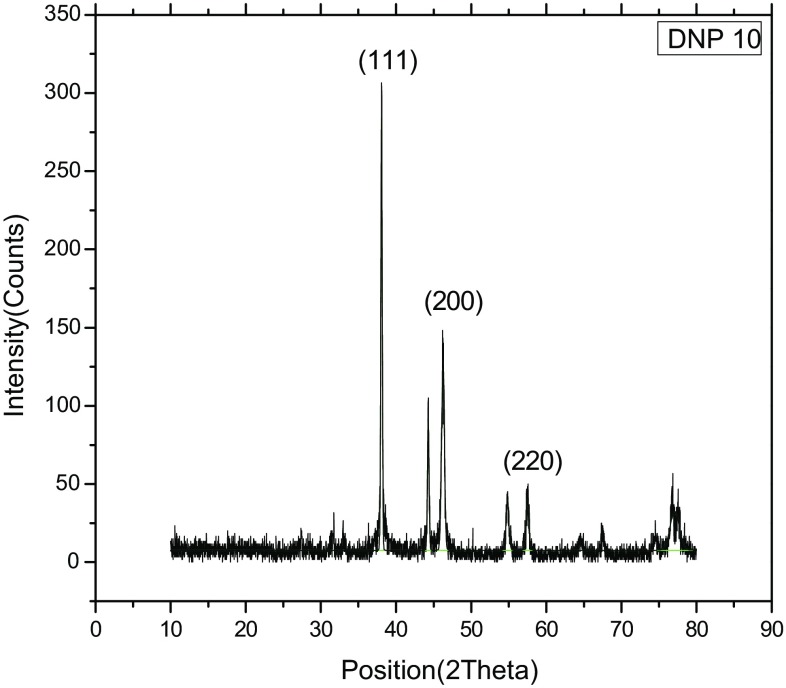
XRD analysis of biologically synthesized AgNPs

**Fig. 4:**
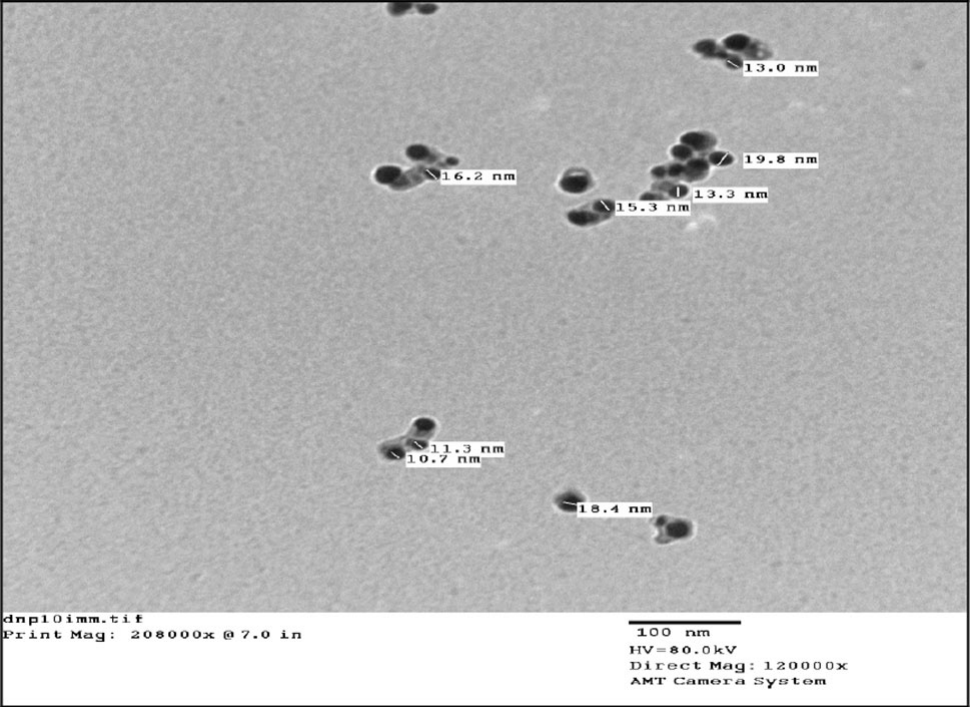
TEM Analysis of biologically synthesized silver nanoparticles by bacterial isolate DNP 10

**Fig. 5:**
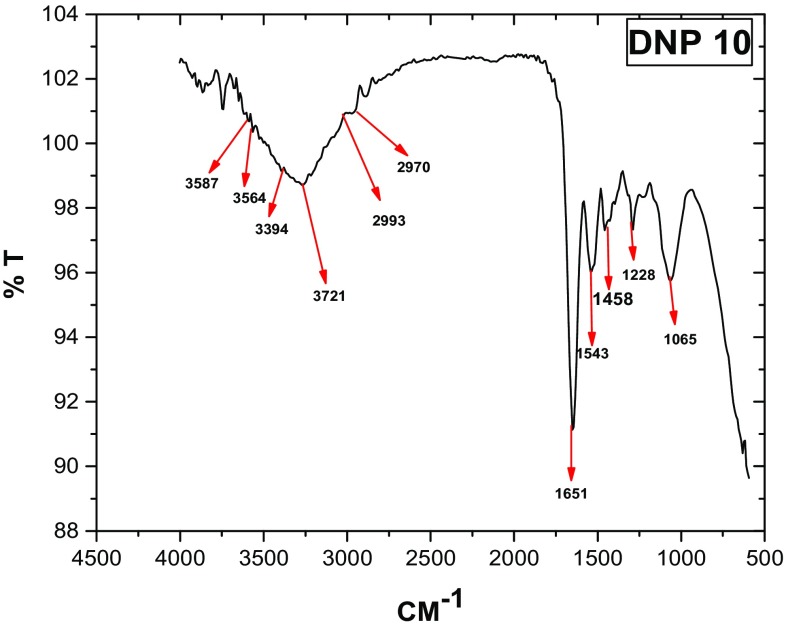
FTIR analysis of biologically synthesized silver nanoparticles

**Fig. 6:**
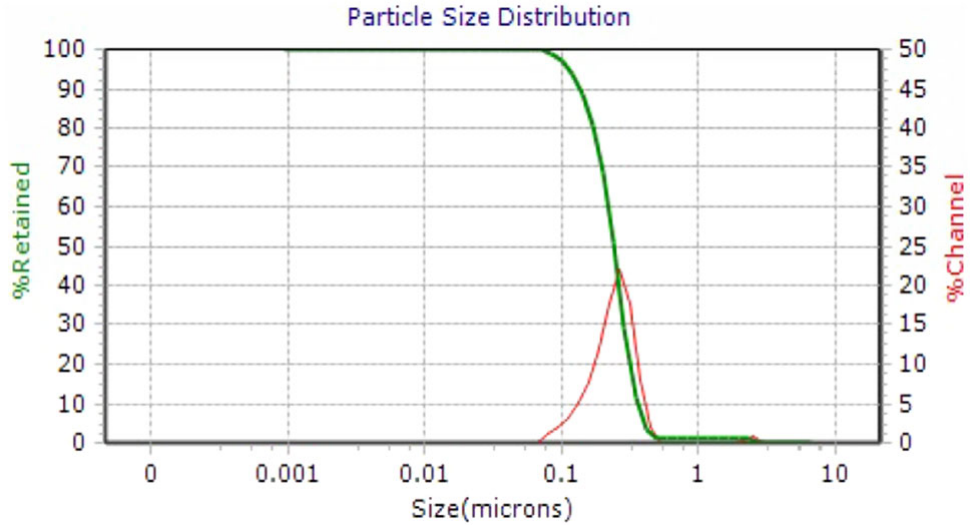
DLS Analysis of biologically synthesized silver nanoparticles

The crystal structure of biologically synthesized silver nanoparticles was determined by X-ray diffraction (XRD) analysis. The XRD pattern shows four intense peaks in the whole spectrum of 2*θ* values ranging from 20° to 80°. In XRD pattern, three diffractions peaks were observed at 38.07, 46.24, 54.82, and 77.110 corresponding to 111, 200, 220, and 311 face centered cubic (fcc) silver planes for metallic silver, respectively as shown in Fig. 3. The size of silver nanoparticles was calculated by using Debye-Scherer’s equation as shown in Table 1. The calculated average size of silver nanoparticles was about 27.25 nm. The calculated size was consistent with the nanoparticle size estimated by TEM analysis. The size and shape of the synthesized silver nanoparticles was determined by TEM analysis. In the TEM analysis, silver nanoparticles were spherical in shape with size ranging from 10 to 30 nm as shown in Fig. 4. The FTIR analysis was carried out to identify possible interactions between silver salts and protein molecules responsible for the reduction of Ag^+^ ions and stabilization of AgNPs. The FTIR spectrum of silver nanoparticles between the wave number 400^−1^–4000^−1^ showed strong peaks at 1651, 1528, 1288, and 1057 cm^−1^ as shown in Fig. 5. The DLS graph of biologically synthesized AgNPs is shown in Fig. 6. The size of AgNPs was around 200–250 nm. The particles carry a charge of **−**39.86 mV. This confirms that the biologically synthesized silver nanoparticles were stable.

**Table 1:** Size calculated by Debye Scherrer equation

Position (2*θ*)	Width	Size (nm)
38.07	0.220	40.53
46.24	0.379	24.23
54.829	0.380	33.79
77.110	1.236	10.460

**Fig. 7:**
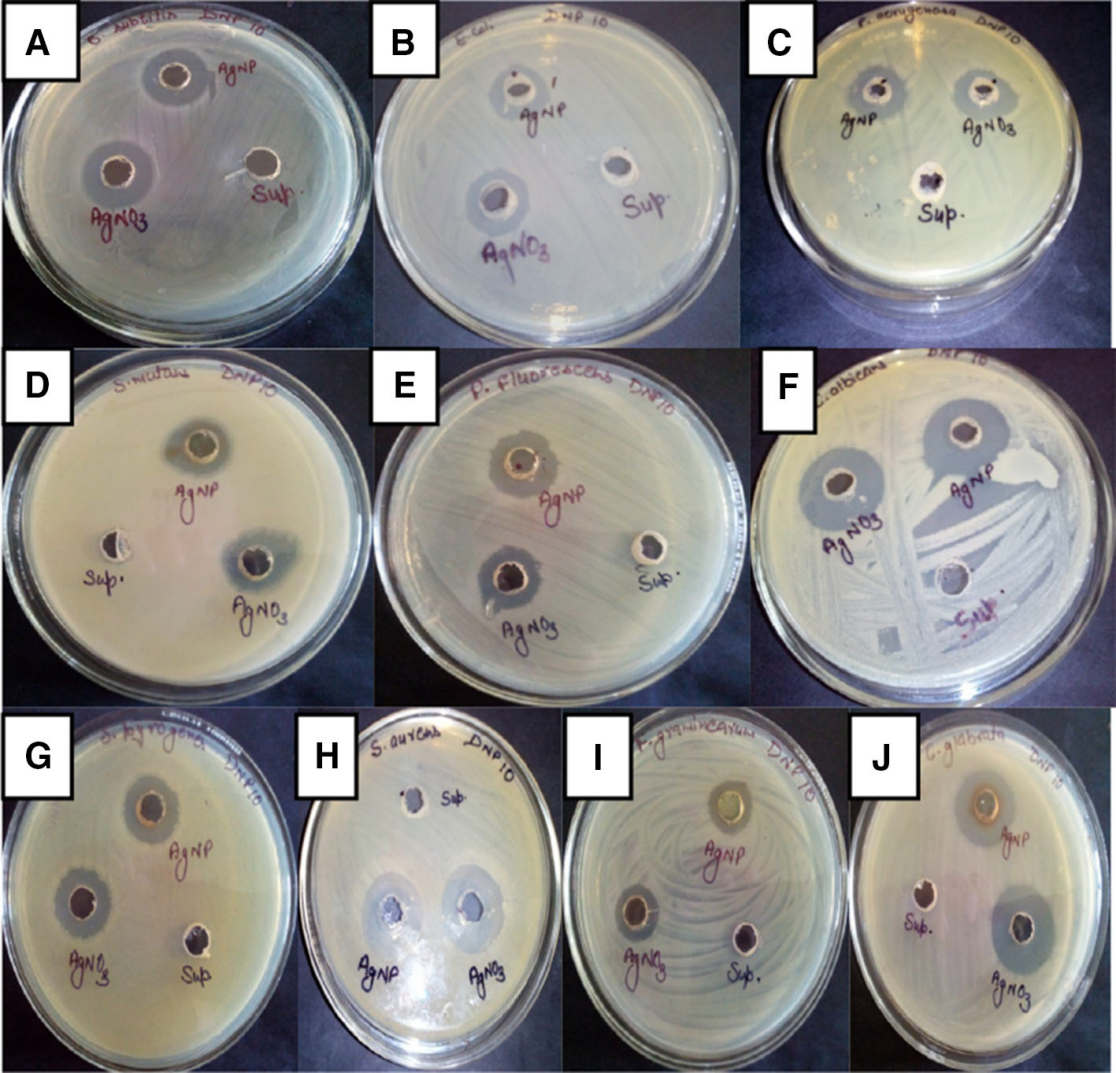
Antimicrobial activity of biologically synthesized AgNP against pathogenic microorganisms **a** *Bacillus subtilis*, **b** *Escherichia coli*, **c** *Pseudomonas aeruginosa*, **d** *Streptococcus mutans*, **e** *Pseudomonas fluorescens*, **f** *Candida albicans*, **g** *Streptococcus pyogenes*, **h** *Staphylococcus aureus*, **i** *Fusarium graminearum*, and **j** *Candida glabrata*

**Fig. 8:**
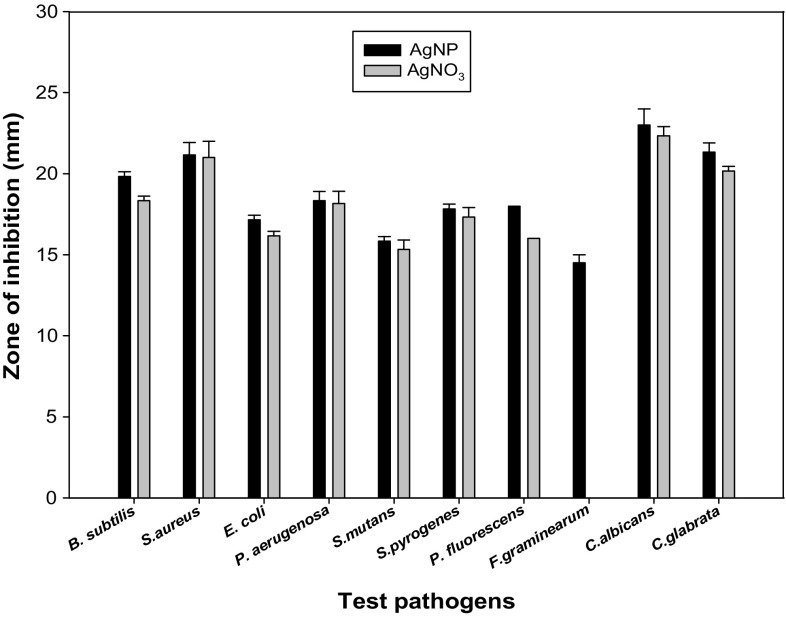
Antimicrobial activity of silver nanoparticles (in mm)

The antibacterial activity of biosynthesized silver nanoparticles was checked against Gram-positive bacteria (*Bacillus subtilis, S. aureus*, *S. mutans*, *S. pyogenes*), Gram-negative bacteria (*E. coli, P. aeruginosa, P. fluorescens*), fungi (*C. albicans*, *C. glabrata*) and plant pathogen (*F. graminearum*). The AgNPs synthesized by isolate *L. varians* showed excellent antibacterial activity against all tested pathogens as shown in Fig. 7. The highest antimicrobial activity of silver nanoparticles synthesized by *L. varians* was found against *C. albicans* (23 mm) and *C. glabrata* (21.33 mm). The lowest antimicrobial activity of biologically synthesized silver nanoparticles was found against *F. graminearum* (14 mm) as shown in Fig. 8. However, antimicrobial activity of silver nanoparticles was significantly higher than silver nitrate in case of *E. coli* and *P. fluorescens*. The MIC values of AgNPs for the selected strains were calculated and summarized in Table 2. The MIC of silver nanoparticles was observed as 46 μg for *E. coli, P. aeruginosa*, *Bacillus subtilis, S. aureus* and 23 μg for *C. albicans*.

**Table 2:** MIC of silver nanoparticles by agar well diffusion assay

Zone of inhibition (mm)					
Test pathogen	Silver nanoparticles concentration (in μg)				
	375	187.5	93.7	46.85	23.42
*B. subtilis*	20	19	18	15	0
*S. aureus*	21	20	19	14	0
*E. coli*	19	18	17	14	0
*P. aeruginosa*	18	16	15	12	0
*C. albicans*	23	21.5	20	19	15

## Discussion

The bacterial mediated synthesis of silver nanoparticles is an attractive and alternative to chemically and physically produced AgNPs due to their green properties. The biological methods of nanoparticle synthesis are economical, easy to perform and environment friendly. Silver nanoparticle had drawn attention because of their extensive application to new technologies in chemistry, electronics, medicine and biotechnology. In the present study, the selected bacterial strain DNP 10 identified as *L. varians* showed ability to synthesize silver nanoparticles by extracellular mechanisms. The screening of microbial isolate for silver nanoparticle synthesis is generally carried out on the basis of colour change (Kalimuthu et al. 2008). Appearance of a dark-brown colour in the solution is a primary indication for AgNPs formation in the reaction mixture (Sastry et al. 2003). The excitation of surface plasmon vibration in silver nanoparticles was considered as the basis for formation of brown colour. Similar observation was previously reported in various studies, where a pale yellow to brown colour was formed due to the reduction of aqueous silver ions to silver nanoparticles (Saravanan et al. 2011). Silver nanoparticles synthesis has also been reported by using *Lysinibacillus sphaericus* MR-1 (Gou et al. 2015) and *Lysinibacillus fusiformis* (Yousef 2014). The extracellular synthesis of silver nanoparticles by using culture supernatant of various bacteria *B. licheniformis* (Kalishwaralal et al. 2008), *Pseudomonas aeruginosa* (Jeevan et al. 2012), *Lactobacillus* sp. (Ranganath et al. 2012), *Exiguobacterium* sp. KNU1 (Dhawal and Dae 2013) and probiotic bacteria *B. Linens* (Ranganathan and Ramachandran 2012) has already been reported. This supports that colour change can be considered as an indication of silver nanoparticles formation. The synthesis process adopted in this study was much faster than previously reported bioreduction processes, which requires hours or days (Vivekanandhan et al. 2014; Song et al. 2009). Synthesis of silver nanoparticles was further confirmed by UV–Vis spectroscopy, which measures the absorption spectra of silver nanoparticles formed due to collective excitation of conduction electrons in the metal. Thus, methods based on UV–Vis spectroscopy have been shown to be an effective technique for the analysis of nanoparticles (Sastry et al. 1998). This is already reported that spherical silver nanoparticles exhibit maximum absorbance between 420 and 450 nm (Ninganagouda et al. 2013; Sunkar and Nachiyar 2012). The size, shape, morphology, composition and dielectric environment of prepared nanoparticles affect the width of SPR bands (Kelly et al. 2003).

The XRD spectrum of synthesized nanoparticles was in agreement with diffraction standard JCPDS 04-0783, which confirms the presence of elemental silver. The XRD peaks at 2*θ* of 38.07, 46.24, 54.82 and 77.110 could be attributed to 111, 200, 220 and 311 crystallographic planes of face-centered cubic silver crystals, respectively. Thus, XRD pattern clearly confirms the crystalline nature of Ag-NPs synthesized in this study. As per XRD analysis in the absence of PVP, silver oxide nanoparticles were formed instead of silver nanoparticles. The PVP was added to stabilize the formation of silver nanoparticles. So, it can be inferred that the extracellular protein present in culture supernatant were responsible for the reduction of silver nitrate and PVP played a major role in the stabilization of formed silver nanoparticles.

TEM analysis of purified silver nanoparticles confirmed that nanoparticles were spherical and also not aggregated. Protein secreted by microorganisms may be responsible for the stability of silver nanoparticles. The size of silver nanoparticles produced by *L. varians* (10–20 nm) fall closer to the size of silver nanoparticles produced by other bacteria (Gurunathan et al. 2009). The silver nanoparticles produced extracellularly by using probiotic bacteria *Lactobacillus* sp. were also in size range of 2–20 nm (Ranganath et al. 2012). The size of nanoparticles can have direct effect on its physico-chemical properties and this can vary among the particles formed by different groups of microorganisms.

The peaks observed at wave number 1651 cm^−1^ indicated the stretching of C = O amide I bands of peptide linkage and 1288 cm^−1^ indicated stretching of CN amines and NH bending of peptide. The peak at 1057 cm^−1^ was due to stretching of C–OH in primary alcohols. This is reported that the carbonyl group (C = O) of amino acid residues and peptides has the ability to bind silver ion (Balaji et al. 2009). The FTIR spectrum indicated that proteins were responsible for stabilizing the silver nanoparticles. DLS measures the hydrodynamic size of silver nanoparticles. The size measured by DLS is not only the size of metallic nanoparticles but it also includes the stabilizers absorbed on the surface of nanoparticles. Thus the size measured by DLS is larger than measured by other techniques. The particles carry a charge of −44.55 mV. This confirms that the biologically synthesized silver nanoparticles are stable. A minimum of ±30 mV is an indication of stability of silver nanoparticles (Jacobs and Muller 2002). The polydispersity index (PdI) of silver nanoparticles was below 0.3.

The antimicrobial activity of silver nanoparticles has been reported against fungus, yeast, microbial biofilms yeast, Gram-negative and Gram-positive bacteria (Kim et al. 2007; Sondi and Salopek-Sondi 2004; Abdeen et al. 2014; Gaidhani et al. 2013; Singh et al. 2013). Antimicrobial activity of silver nanoparticles has been reported against both Gram-positive as well as Gram-negative bacteria (Sondi and Salopek-Sondi 2004). The antimicrobial activity of synthesized nanoparticles was reported against *P. aeruginosa, S. aureus, Aspergillus flavus* and *Aspergillus niger* (Govindaraju et al. 2010; Mirzajani et al. 2011). AgNPs synthesized by isolated bacterial strain DNP 10 (*L. varians*) showed excellent antibacterial activity against all tested strains (bacterial and fingal). In this study, MIC of PVP coated silver nanoparticles against *E. coli*, *S. aureus*, *B*. *subtilis*, *P*. *aeruginosa* was evaluated as 46 μg/mL.MIC of silver nanoparticles against *C. albicans* was evaluated as 23 μg/mL. Various studies have reported different values of MIC for different pathogens. Shrivastava et al. (2009) determined the average MIC value of AgNPs against *E. coli* as 25 and 100 μg/mL MIC value against *S. aureus*. However, Dos Santos et al. (2014) reported MIC value as 100 μg/mL for both *E. coli* and *S. aureus*. MIC of silver nanoparticles against *P. aeruginosa* has been reported as 75 μg/mL (Morones et al. 2005) and 20 μg/mL (Palanisamy et al. 2014) in two different studies. MIC of silver nanoparticles against *B. subtilis* is reported as 40 μg/mL (Ruparelia et al. 2008). Different MIC values for same pathogens may be due to difference in strains of pathogens, source of pathogens and different size and shape of nanoparticles. Stabilization method employed for synthesized nanoparticles also influenced the efficiency of silver nanoparticles. MIC of citrate capped silver nanoparticles has been reported as 5–10 μg/mL against *E. coli* (Zhou et al. 2012). Polyvinylpyrrolidone (PVP) showed good antibacterial activity towards *S. aureus*, *E. coli*, *P. aeruginosa*, *Bacillus subtilis* and good fungicidal activity against various yeasts and molds (Bryaskova et al. 2011). The antimicrobial activity of AgNPs is inversely related to size and shape (Pal et al. 2007). The high specific surface-to-volume ratio of silver nanoparticles increases their contact with microorganisms, promoting the dissolution of silver ions, thereby improving biocidal effectiveness. The ability of silver nanoparticles to release silver ions is a key to their bactericidal activity (Stobie et al. 2008).

## Conclusion

In conclusion, *L. varians* possess an ability to synthesize silver nanoparticles by extracellular method. The process adopted for nanoparticles synthesis was simple, fast, environment friendly and easy to perform. The biologically synthesized PVP coated silver nanoparticles were stable and well dispersed. The synthesized silver nanoparticles showed antimicrobial activity against bacteria, fungi and plant pathogen. This study showed that biologically synthesized Ag-NPs have great potential of antimicrobial agent and can have application in various fields.
